# Erythrocyte Sedimentation Rate as a Monitoring Marker in the Canine Intensive Care Unit

**DOI:** 10.1111/vec.70058

**Published:** 2026-01-30

**Authors:** Eleonora Gori, Anna Pasquini, Angela Briganti, Daniela Diamanti, Veronica Marchetti

**Affiliations:** ^1^ Veterinary Teaching Hospital “Mario Modenato”, Department of Veterinary Sciences University of Pisa Pisa Italy; ^2^ DIESSE Diagnostica Senese Spa Monteriggioni Italy

**Keywords:** dog, erythrocyte sedimentation rate, mortality, POCT, sepsis

## Abstract

**Objective:**

To (1) establish whether the erythrocyte sedimentation rate (ESR) at admission is related to mortality in dogs hospitalized in the ICU; (2) observe and evaluate the ESR trend during 48–72 h of hospitalization and determine how it relates to mortality; and (3) test whether ESR is a marker of sepsis.

**Design:**

Prospective study using residual K3‐EDTA blood samples of hospitalized dogs.

**Setting:**

ICU of a university teaching hospital.

**Animals:**

A total of 124 hospitalized dogs were included in the study. Sixty of the 124 dogs were used to test whether ESR is a marker of sepsis.

**Measurements and Main Results:**

The ESR was measured on residual EDTA blood samples collected from hospitalized dogs as part of clinical evaluation. A total of 32 dogs died during hospitalization, while 92 were discharged. The ESR at admission (T0) was significantly higher in nonsurvivors (28 mm/h) compared with survivors (11 mm/h) (*p* = 0.03). Forty‐one dogs had ESR monitored at T1 (24 h postadmission) and T2 (48–72 h postadmission). An increase in the ESR from T0 to T2 was seen in nonsurvivors (*p* < 0.01; medians: T0, 22 mm/h, T1, 37 mm/h, T2, 42 mm/h). Survivors showed a decrease in the ESR from T0 to T2 (*p* < 0.01; medians: T0, 12 mm/h, T1, 14 mm/h, T2, 5 mm/h). Twenty‐eight dogs were diagnosed with sepsis and had a higher ESR than nonseptic dogs (35 vs. 10 mm/h; *p* < 0.0001). A cutoff of 22 mm/h may differentiate septic dogs from nonseptic dogs, with a sensitivity of 76% and a specificity of 81% (area under the curve = 0.8; *p* < 0.0001).

**Conclusions:**

The ESR at admission can predict the mortality of hospitalized dogs. Its monitoring during hospitalization may add prognostic information. Given the challenges involved in screening septic patients, point‐of‐care testing may easily evaluate the ESR when used alongside other indicators.

**Abbreviations**
AUC: area under the curve
ESR: erythrocyte sedimentation rate
IQR: interquartile range
ROC: receiver operating characteristic

## 1 Introduction

The erythrocyte sedimentation rate (ESR) has been correlated with major canine acute phase proteins, such as C‐reactive protein and fibrinogen [1, 2, 3]. It has been tested as an inflammatory marker and monitoring parameter in various conditions, including canine babesiosis, ehrlichiosis [4, 5], and canine osteoarthritis [6].

Automated continuous‐loading instruments have been used in recent studies to measure the ESR. This technique has been compared with the gold standard Westergren method and has been validated in dogs [7]. The main advantages of a semiautomatic ESR reader^1^ include its fast turnaround time (14 min) and the use of routine K‐EDTA blood tubes [7]. The canine ESR value remains stable for 6 h at room temperature or 24 h if refrigerated at 4°C ± 2°C [8]. The device is not operator dependent, and results are displayed on the device screen.

The ESR has been studied in various scenarios in both pediatric [9, 10, 11, 12] and adult ICU patients [13, 14, 15, 16, 17, 18]. In children, the ESR has been shown to be a biomarker of neonatal sepsis and inflammation. It is higher in children with sepsis but performs poorly in terms of predictability [9, 10, 11, 12]. In adults, a moderate‐to‐marked increase in ESR is associated with a higher risk of mortality [15], and increased ESRs have been independently associated with mortality in ICU patients [13]. During the COVID‐19 pandemic, the ESR differentiated between severe and nonsevere cases, serving as an independent prognostic indicator of disease severity and mortality [16, 17, 18].

Before the introduction and validation of the abovementioned semiautomatic ESR reader^a^, the clinical application of the ESR in dogs was abandoned due to long processing time and the use of other acute phase proteins. The automated device in the current study, however, has revived interest in the clinical application of the ESR in canine emergency and critical care medicine. The objectives of our study were to establish whether the ESR at admission relates to mortality in hospitalized dogs; evaluate the ESR in hospitalized dogs at admission (T0) and during hospitalization for 48–72 h, observing its trend and how it relates to mortality; and test whether the ESR at admission is associated with a diagnosis of sepsis.

## 2 Materials and Methods

### 2.1 Study Design and Population Selection

This prospective study on residual K3‐EDTA blood samples of hospitalized dogs was conducted at the University of Pisa Veterinary Teaching Hospital between September 2021 and February 2024. Formal ethical approval was not required because residual blood samples were used. All owners provided informed consent for use of their pet's residual blood samples.

ESR measurement was performed on each hospitalized dog that had previously provided a blood sample for routine care and monitoring. Signalment, clinical history, clinical examination, and outcome were recorded for each dog. Based on discharge outcome, dogs were divided into survivor and nonsurvivor groups. Dogs were classified as survivors if they were discharged alive; otherwise, they were classified as nonsurvivors.

For the first objective of our study, the ESR at admission (T0) was analyzed in relation to mortality. For the second objective, dogs with serial CBCs were included. Using the residual sample from each respective CBC, the ESR was evaluated at the time of admission (T0) and at 24–36 and 48–72 h after admission (T1 and T2, respectively). For our third objective, we used the criteria for systemic inflammatory response syndrome to define sepsis as long as an infectious underlying cause (i.e., positive bacterial culture or cytologic evidence of bacteria) was documented. The criteria for systemic inflammatory response syndrome included at least two of the following four: temperature <38.1°C or >39.2°C; heart rate >120/min; respiratory rate >20/min; and total WBC count >16 × 10^9^/L, <6 × 10^9^/L, or band neutrophils >3% of WBC count [19, 20].

### 2.2 ESR Measurement

Following the manufacturer's instructions, the semiautomatic ESR reader^a^, a point‐of‐care testing device with four channels containing tube holders and optical units, was used to measure the ESR [7]. The same blood tube used for CBC evaluation^2^ was used, with measurements performed immediately after CBC processing.

EDTA blood was mixed 10 times by gentle inversion and placed into one of the four channels. The blood tube was then scanned by the corresponding optical unit. The operator set the species of the sample. After 14 min of optical reading, the ESR result (mm/h) was displayed on the screen [7]. If an “error” message appeared, the blood tube was removed from the device and gently mixed 10 times as described above, and the test was repeated [7, 8]. If the measurement failed three times, the sample was classified as “not measurable” and was excluded from analysis.

### 2.3 Statistical Analysis

All analyses were performed using two commercial statistical software packages^3^^,^ 4. Continuous variables are reported as medians and 25th to 75th percentiles (interquartile range, IQR) if nonnormally distributed. Normally distributed variables are reported as mean ± SD.

For the first study objective, the Mann–Whitney *U*‐test was used to compare the T0 ESR of survivors and nonsurvivors. T0, T1, and T2 ESRs of survivors and nonsurvivors were compared with the Friedman test for our second objective. Durbin–Conover pairwise comparisons were also performed. For the third objective, the T0 ESR of septic and nonseptic dogs was compared using the Mann–Whitney *U*‐test.

A receiver operating characteristic (ROC) curve was constructed to find the optimal ESR cutoff for the presence of sepsis in ICU patients. The area under the curve (AUC) was calculated, and the value with the best sensitivity and specificity was chosen as the cutoff. A *p*‐value of <0.05 was considered statistically significant.

## 3 Results

### 3.1 Study Population

**FIGURE 1:**
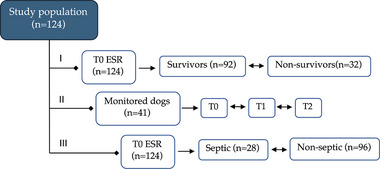
Flowchart of the study design showing our objectives to (1) establish whether ESR at admission relates to mortality in hospitalized dogs (92 survivors vs. 32 nonsurvivors); (2) evaluate the ESR in 41 hospitalized dogs at admission (T0) and during hospitalization for 48 (T1) to 72 h (T2) to observe its trend and how it relates to mortality; and (3) test whether the ESR at admission is associated with a diagnosis of sepsis. Each horizontal line represents an aim of the study. ESR, erythrocyte sedimentation rate; T, time.

A total of 124 hospitalized dogs were included in this study (Figure 1). The most common dog breed was mixed breed (*n* = 37), with the remaining 87 belonging to various breeds. Cocker Spaniel, Golden Retriever, Labrador Retriever, and German Shepherd Dog were the most commonly represented pure breeds (*n* = 6 for each breed). Akita, Dachshund, English Setter, Poodle, and Weimaraner (*n* = 4 each breed) were also common. Fifty‐eight dogs were female, 25 of which were neutered, and 66 were male, 11 of which were neutered. The median age of the dogs was 8 years (IQR: 3.1–11.4 years).

### 3.2 Relationship Between Admission ESR and Overall Mortality of Hospitalized Dogs

**FIGURE 2:**
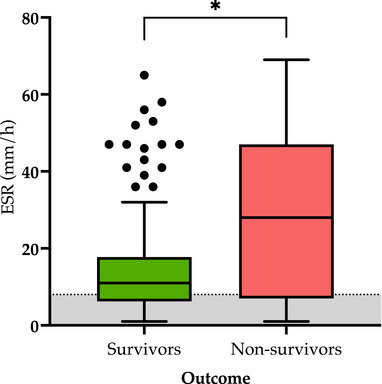
Box‐and‐whisker plot of the admission (T0) ESR in survivors (*n* = 92; S, light green) and nonsurvivors (*n* = 32; NS, light red). The line within the box represents the median value. The gray area shows the reference interval for canine ESR. *p = 0.03. ESR, erythrocyte sedimentation rate.

Of the 124 dogs included to address the first objective of the study, 32 (26%) died during hospitalization, while 92 survived (74%). The ESR at admission (T0) was higher in nonsurvivors (28 mm/h [IQR: 7–47 mm/h]) than in survivors (11 mm/h [IQR: 6–17 mm/h]; *p* = 0.03) (Figure 2).

### 3.3 ESR Trend in Hospitalized Dogs and Its Relation to Mortality

**FIGURE 3:**
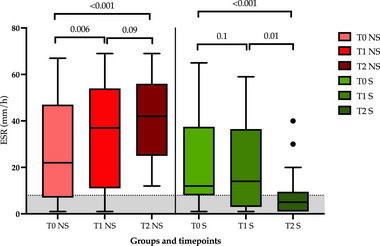
Box‐and‐whisker plot comparing the ESR at different timepoints in nonsurvivors (red) and survivors (green). The line within the boxes represents median values. The gray area shows the reference interval for canine ESR. ESR, erythrocyte sedimentation rate; NS, nonsurvivors; S, survivors; T0, admission; T1, 24 h after admission; T2, 48–72 h after admission.

Sixty hospitalized dogs met the inclusion criteria for the second study objective. The individual demographics and clinicopathological data points of this population are shown in Table S1. Nineteen dogs (32%) died during hospitalization and were classified as nonsurvivors. Eight of these dogs were euthanized for rapid deterioration in clinical condition. The remaining 41 dogs (68%) were discharged alive and thus classified as survivors. As shown in Figure 3, an increase in ESR was seen in nonsurvivors from T0 to T1 (*p* = 0.006) and from T0 to T2 (*p* < 0.001; medians: T0, 22 mm/h, T1, 37 mm/h, and T2, 42 mm/h, respectively), while a decrease was seen in survivors from T0 to T2 (*p* < 0.0001) and from T1 to T2 (*p* = 0.01; medians: T0, 12 mm/h, T1, 14 mm/h, and T2, 5 mm/h, respectively).

### 3.4 Evaluation of the ESR as a Possible Predictor of Sepsis in Hospitalized Dogs

**FIGURE 4:**
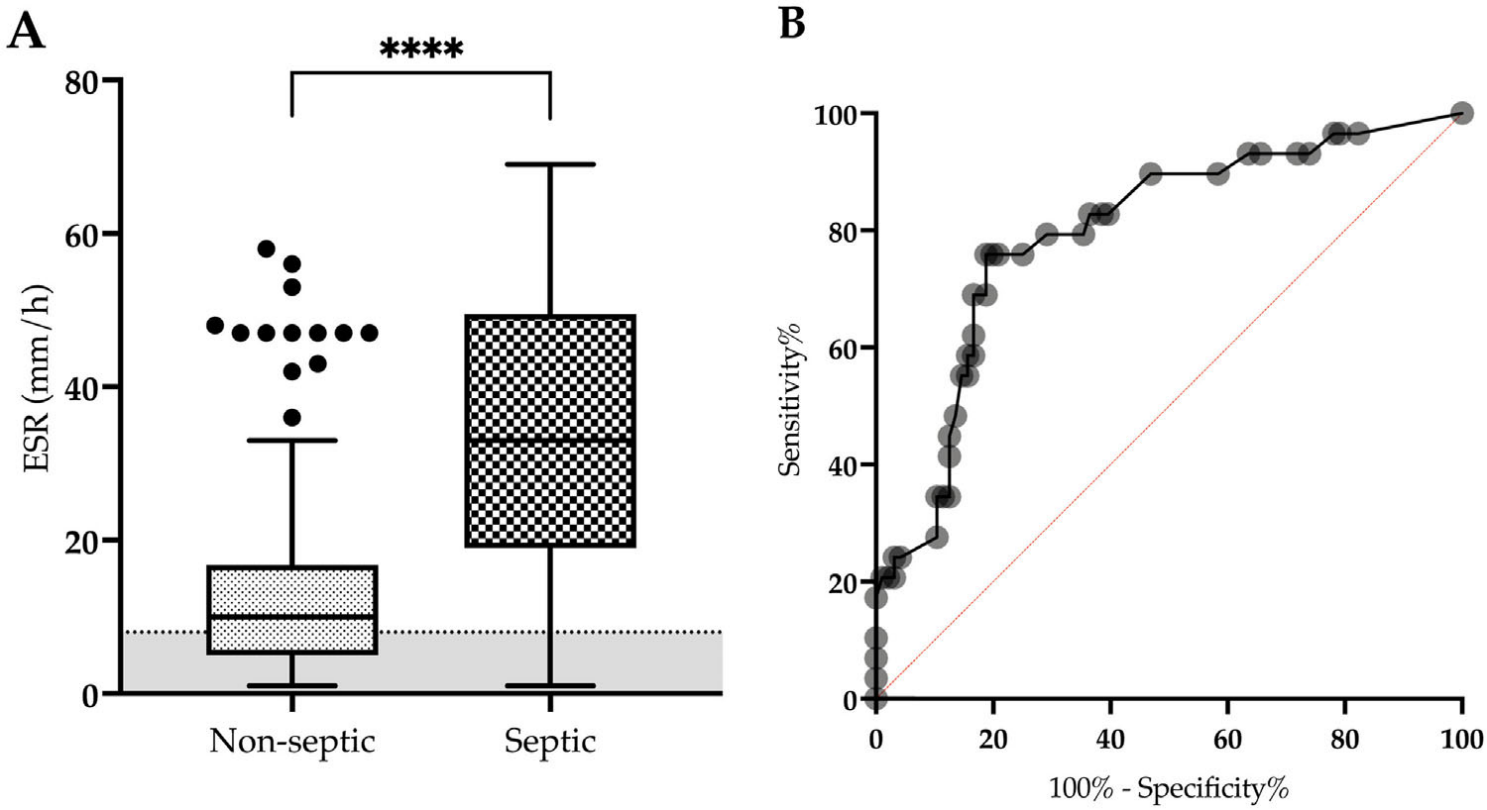
(A) Box‐and‐whisker plot of the admission (T0) ESR in nonseptic (*n* = 96) and septic dogs (*n* = 28). The gray area shows the reference interval for canine ESR (range: 1–8 mm/h). ****p < 0.0001. (B) Receiver operating characteristic curve of the ESR in nonseptic and septic dogs. ESR, erythrocyte sedimentation rate.

For our study's third objective, 28 dogs (19.7%) met the criteria for sepsis. Dogs with sepsis had a higher ESR than nonseptic dogs (35 mm/h [IQR: 22–48 mm/h] vs. 10 mm/h [IQR: 5–16 mm/h], respectively; *p* < 0.0001) (Figure 4A). Based on the ROC curve, the best cutoff for the ESR was 22 mm/h. This cutoff had a sensitivity of 76% and a specificity of 81% for differentiating between septic dogs and nonseptic dogs (AUC = 0.8; *p* < 0.0001) (Figure 4B).

## 4 Discussion

Our study found that the ESR at admission was associated with mortality and sepsis in hospitalized dogs. The ESR measures the rate at which RBCs sediment in a vertical tube over 1 h. Normally, RBCs repel each other due to negative surface charges [21]. Plasma proteins can neutralize these charges, causing the formation of rouleaux and increasing the ESR, which makes RBCs fall faster under inflammatory conditions [1, 2, 21]. The ESR is not specific to any disease but can indicate general inflammation [8]. Its stability, reproducibility, and low cost make it useful as a tool to evaluate illness severity in dogs. Recent studies have focused on validating automatic optical readers for ESR measurements [1, 2, 3, 7, 22]. The semiautomatic ESR reader^a^ device used in the current study allows measurement of the ESR in emergency and intensive care settings. It is simple to use and returns results quickly (14 min vs. 1 h needed for the Westergren method). EDTA samples are used, which prevents excess blood sampling, whereas 2 mL of sodium‐citrate blood is needed for the Westergren method [23].

The results of our study found higher ESRs in nonsurvivors than in survivors. This aligns with studies in people, in whom higher ESR values predict ICU mortality [13]. A previous study reported that a moderate‐to‐marked increase in ESR is associated with a significant risk of overall mortality [15]. In our study specifically, a moderate and marked elevated ESR appeared to result in a two‐ to threefold higher risk of mortality, respectively. These data can easily be explained because the ESR in both people and dogs acts as a nonspecific marker of inflammation and is mostly connected to increases in fibrinogen [1, 2, 21]. Thus, the association between faster ESR and mortality implies that nonsurvivors undergo more severe inflammatory processes.

We demonstrated that not only is the ESR at admission useful, but its trend during hospitalization may also be informative. In fact, survivors had a reduction in the ESR at T1 and T2 (48–72 h after admission), with most dogs returning to the reference interval, while an increase in the ESR was seen in nonsurvivors throughout our testing. To the best of our knowledge, these data can only be compared with human studies on ESR trends because they have never been used in veterinary medicine. In people with osteomyelitis, an initial ESR and first 4‐week ESR trend help to predict treatment duration and recurrence. Based on specific ESR trends, clinicians can determine which patients are likely to require prolonged treatment or have a high risk for osteomyelitis recurrence [24].

Unlike other acute phase proteins such as C‐reactive protein, which increases within 4–6 h of the beginning of inflammatory stimulus, the ESR begins its rise within 24–48 h of the onset of inflammation, decreases slowly as inflammation resolves, and can take weeks to completely normalize [25]. Thus, it is probably more suitable for clinical monitoring, following the course of disease over time, and for predicting treatment response and duration for various inflammatory diseases [25].

Last, our study found higher ESRs in dogs with sepsis, which may help distinguish septic dogs from nonseptic dogs. This finding mirrors those in cases of pediatric and neonatal sepsis. The ESR has been used in neonatal sepsis, where a result of >15 mm/h strongly suggests infection [10]. However, some studies in pediatric medicine have shown mixed results regarding the power of the ESR to predict sepsis. In a systematic review and meta‐analysis of biomarkers that detect neonatal sepsis in low‐ and middle‐income countries, an ESR ≥15 mm/h had a low Youden's index and poor discriminatory value. Blood cultures were used as the reference standard [12].

The current study has limitations. The underlying diseases in the study population were not fully characterized. It would be interesting to investigate the diagnostic/prognostic ability of the ESR in specific diseases or systems, as would a comparison of the ESR with disease severity scales. However, due to the observational nature of this study, some parameters were not available for some of the dogs. A further area for investigation would be the integration of ESR assessment into sepsis severity scales, given the findings of this study that suggest its usefulness as a possible marker for sepsis. In addition, we chose to look at the ESR specifically in relation to the presence of sepsis because it is a very common and often fatal clinical entity for which there are still not enough markers to help clinicians diagnose and monitor sepsis.

In conclusion, the ESR at admission may predict mortality in hospitalized dogs, and its monitoring may provide additional prognostic information during hospitalization. Given that identifying septic patients can be difficult, evaluation of the ESR with a novel semiautomatic reader may be a helpful addition to the diagnostic battery.

## Author Contributions

**Eleonora Gori**: conceptualization, data curation, formal analysis, writing – original draft. **Anna Pasquini**: data curation, writing – review and editing. **Angela Briganti**: formal analysis, writing – review and editing. **Daniela Diamanti**: conceptualization, writing – review and editing. **Veronica Marchetti**: conceptualization, formal analysis, writing – review and editing.

## Conflicts of Interest

Dr. Daniela Diamanti is a DIESSE employee. The other authors declare no conflicts of interest.

## Supporting information




**Supplementary File 1**: vec70058‐sup‐0001‐tableS1.docx

## Endnotes

1 MINI‐PET, DIESSE Diagnostica Senese S.p.A., Monteriggioni, Italy.

2 Idexx Procyte Dx, Idexx Laboratories, Milan, Italy.

3 GraphPad Prism 10 for macOS, GraphPad Software, US.

4 The jamovi project (2023). jamovi (Version 2.3). Retrieved from https://www.jamovi.org.
