# Supplementary material for: Erythrocyte Sedimentation Rate as a Monitoring Marker in the Canine Intensive Care Unit
Source: J Vet Emerg Crit Care (San Antonio). 2026 Jan 30;36(1):33–8. doi: 10.1111/vec.70058 (PMC12950941; doi:10.1111/vec.70058)
Supplement: Supplementary file 1 — vec70058‐sup‐0001‐tableS1.docx [file VEC-36-33-s001.docx]

| Case# | Sex | Breed | Age  (years) | Outcome | Diagnosis | T0 ESR  (mm/h) | T1 ESR  (mm/h) | T2 ESR  (mm/h) |
| --- | --- | --- | --- | --- | --- | --- | --- | --- |
| 1 | M | Mix Breed | 3,1 | NS | Extrahepatic biliary tract obstruction, sepsis | 33 | 48 | 51 |
| 2 | F | Weimaraner | 14,9 | NS* | Pyometra, sepsis | 67 | 69 | 69 |
| 3 | F | Weimaraner | 4,9 | NS* | Glossitis, sepsis | 22 | 29 | 29 |
| 4 | M | Cocker Spaniel | 8,1 | NS | Gastrointestinal foreign body | 13 | 25 | 25 |
| 5 | M | Mix Breed | 11,1 | NS | Chronic kidney disease | 47 | 1 | 63 |
| 6 | FS | Mix Breed | 6,0 | NS* | Uncontrolled epilepsy, Liver disease | 7 | 42 | 42 |
| 7 | M | Dobermann Pinscher | 6,4 | NS | Aspiration pneumonia | 7 | 37 | 37 |
| 8 | FS | Akita Inu | 6,7 | NS | Post-chemo sepsis | 65 | 68 | 68 |
| 9 | MC | Jack Russel Terrier | 16,9 | NS* | Acute kidney injury | 2 | 12 | 17 |
| 10 | MI | Golden Retriever | 4,4 | NS | Aspiration pneumonia | 1 | 47 | 50 |
| 11 | MI | Mix Breed | 7,5 | NS | Acute-on-chronic kidney injury | 33 | 52 | 52 |
| 12 | MI | Welsh Terrier | 10,5 | NS | Adrenal tumour | 47 | 54 | 54 |
| 13 | FI | Miniature Schnauzer | 13,5 | NS | Pyometra, sepsis | 1 | 11 | 20 |
| 14 | MC | German Shepherd | 12,7 | NS* | Hepatic tumour | 5 | 5 | 12 |
| 15 | M | English Springer Spaniel | 4,3 | NS | Acute kidney injury | 47 | 56 | 56 |
| 16 | M | White Swiss Shepherd Dog | 5,2 | NS* | Acute kidney injury + acute pancreatitis | 10 | 10 | 12 |
| 17 | MI | Poodle | 12,4 | NS* | Vestibular syndrome | 42 | 35 | 35 |
| 18 | FI | Mix Breed | 6,4 | NS* | Diabetic ketoacidosis, sepsis | 9 | 11 | 30 |
| 19 | MI | Mix Breed | 11,4 | NS | Acute kidney injury | 58 | 66 | 66 |
| 20 | M | Cane Corso | 10,1 | S | Chronic kidney disease | 16 | 59 | 1 |
| 21 | F | Australian Shepherd | 11,8 | S | Pyometra | 41 | 52 | 1 |
| 22 | F | Mix Breed | 15,2 | S | Pyometra | 5 | 37 | 1 |
| 23 | MC | English Setter | 4,8 | S | Vertebral abscess | 36 | 9 | 6 |
| 24 | F | Akita Inu | 9,6 | S | Hit-by-car trauma | 12 | 16 | 13 |
| 25 | M | Mix Breed | 11,7 | S | Prostatic abscesses | 15 | 15 | 1 |
| 26 | MI | Bernese Mountain Dog | 3,0 | S | Sepsis | 11 | 9 | 3 |
| 27 | FI | Labrador Retriever | 4,5 | S | Pyometra | 10 | 9 | 6 |
| 28 | MC | Labrador Retriever | 1,3 | S | Splenic hemangiosarcoma | 43 | 28 | 6 |
| 29 | MI | American Staffordshire Terrier | 12,8 | S | Prostatic abscesses | 16 | 13 | 11 |
| 30 | MI | Dachshund | 3,0 | S | Uncontrolled chronic enteropathy | 1 | 1 | 1 |
| 31 | MI | French Bulldog | 3,1 | S | Post-surgical cystotomy | 1 | 1 | 1 |
| 32 | MI | Mix Breed | 0,4 | S | Gallbladder mucocele | 56 | 14 | 1 |
| 33 | FS | Beagle | 2,1 | S | Lymphoma | 6 | 1 | 1 |
| 34 | MC | Mix Breed | 2,4 | S | Prostatic abscesses | 65 | 45 | 10 |
| 35 | MI | Bernese Mountain Dog | 11,4 | S | Chronic enteropathy, pancreatitis | 11 | 12 | 2 |
| 36 | FI | Mix Breed | 10,2 | S | Intestinal foreign body | 10 | 41 | 9 |
| 37 | MI | Staffordshire Bull Terrier | 12,2 | S | ARDS | 11 | 15 | 2 |
| 38 | FI | Kurzhaar | 7,4 | S | Intervertebral foreign body | 27 | 45 | 20 |
| 39 | FS | Cocker Spaniel | 2,8 | S | Acute pancreatitis | 53 | 38 | 11 |
| 40 | FI | Kurzhaar | 0,8 | S | Pyometra | 5 | 1 | 1 |
| 41 | MC | Mix Breed | 2,5 | S | Acute pancreatitis | 10 | 6 | 5 |
| 42 | FS | Dachshund | 3,2 | S | Hepatic abscess, hyperadrenocorticism | 46 | 36 | 15 |
| 43 | FI | Mix Breed | 1,5 | S | Pulmonary angiostrongylosis | 17 | 2 | 2 |
| 44 | FI | Dachshund | 9,7 | S | Pyometra | 52 | 47 | 30 |
| 45 | MI | Malinois Belgian Shepherd | 9,4 | S | Uncontrolled immunomediated thrombocytopenia | 10 | 50 | 40 |
| 46 | MI | Mix Breed | 11,1 | S | Intestinal intussusception | 10 | 30 | 8 |
| 47 | MI | Mix Breed | 8,5 | S | Prostatic abscesses, sepsis | 39 | 43 | 6 |
| 48 | FS | Poodle | 11,8 | S | Acute pancreatitis | 21 | 7 | 7 |
| 49 | MI | Cavalier King Charles Spaniel | 5,6 | S | Acute pancreatitis | 47 | 33 | 12 |
| 50 | MC | Mix Breed | 2,2 | S | Acute enteropathy | 1 | 1 | 1 |
| 51 | FS | Miniature Schnauzer | 12,3 | S | Acute pancreatitis | 32 | 3 | 3 |
| 52 | MC | Dalmatian | 15,0 | S | Uncontrolled epilepsy | 7 | 1 | 1 |
| 53 | F | Mix Breed | 11,4 | S | Pyometra | 11 | 2 | 2 |
| 54 | FS | Labrador Retriever | 13,5 | S | Acute enteropathy | 18 | 16 | 16 |
| 55 | F | Rottweiler | 10,8 | S | Pyometra | 1 | 16 | 1 |
| 56 | MI | Staffordshire Bull Terrier | 10,1 | S | Peripheral vestibular syndrome | 47 | 8 | 8 |
| 57 | MI | Miniature Pinscher | 11,1 | S | Traumatic hemoabdomen | 9 | 6 | 6 |
| 58 | FS | Mix Breed | 1,8 | S | Uncontrolled IMHA, Leishmania+ | 16 | 16 | 9 |
| 59 | MI | Rhodesian Ridgeback | 3,8 | S | Pulmonary angiostrongylosis | 5 | 1 | 1 |
| 60 | FS | Mix Breed | 0,4 | S | Acute pancreatitis | 1 | 3 | 2 |
